# Natural Negative Allosteric Modulators of 5-HT_3_ Receptors

**DOI:** 10.3390/molecules23123186

**Published:** 2018-12-03

**Authors:** Lina T. Al Kury, Mohamed Mahgoub, Frank Christopher Howarth, Murat Oz

**Affiliations:** 1 Department of Health Sciences, College of Natural and Health Sciences, Zayed University, Abu Dhabi 144534, UAE; 2 Departments of Pharmacology, College of Medicine and Health Sciences, UAE University, Al Ain 15551, UAE; mahjoub_mohd@live.com; 3 Departments of Physiology, College of Medicine and Health Sciences, UAE University, Al Ain 15551, UAE; chris.howarth@uaeu.ac.ae; 4 Department of Pharmacology and Therapeutics, Faculty of Pharmacy, Kuwait University, Kuwait City 13060, Kuwait; muratoz1@yahoo.com

**Keywords:** chemotherapy-induced nausea and vomiting, 5-hydroxytryptamine3 receptors, 5-hydroxytryptamine3 receptor antagonists, negative allosteric modulators

## Abstract

Chemotherapy-induced nausea and vomiting (CINV) remain the most common and devastating side-effects associated with cancer chemotherapy. In recent decades, several lines of research emphasize the importance of 5-hydroxytryptamine3 (5-HT_3;_ serotonin) receptors in the pathogenesis and treatment of CINV. 5-HT_3_ receptors are members of ligand-gated ion channels that mediate the rapid and transient membrane-depolarizing effect of 5-HT in the central and peripheral nervous system. These receptors play important roles in nausea and vomiting, as well as regulation of peristalsis and pain transmission. The development of antagonists for 5-HT_3_ receptor dramatically improved the treatment of CINV in cancer patients. In fact, the most common use of 5-HT_3_ receptor antagonists to date is the treatment of nausea and vomiting. In recent years, there has been an increasing tendency to use natural plant products as important therapeutic entities in the treatment of various diseases. In this article, we examined the results of earlier studies on the actions of natural compounds on the functional properties of 5-HT_3_ receptors. It is likely that these natural modulators of 5-HT_3_ receptors can be employed as lead structures for the synthesis of therapeutic agents for treating CINV in future clinical studies.

## 1. Introduction

Chemotherapy-induced nausea and vomiting (CINV) remain the most common and devastating side-effects associated with cancer treatment. These side effects can lead to the development of serious clinical complications such as electrolyte imbalance, dehydration, weight loss, weakness, fractures and deterioration in behavioral and mental status of the patient. In addition, these side effects can interfere with compliance with treatment as patients may delay or refuse future treatments because of fear of further nausea and vomiting [1,2]. Recently, a number of approaches have been applied to control CINV including medicinal or complementary therapies and the selection and prescription of appropriate approaches will significantly improve the patients’ quality of life.

Chemotherapy treatments received in cancer patients are classified into high emetic risk (emesis in nearly all patients), moderate emetic risk (30–90% risk of emesis), low emetic risk and minimal emetic risk emetogenic chemotherapies [3,4,5]. Compared to cisplatin, melphalan, cyclophosphamide and dacarbazine, which have high emetogenic potential, other chemotherapy agents such as anthracyclines, methotrexate, oxaliplatin and carboplatin have moderate emetogenic properties [3,4,5]. Depending on the chemotherapeutic agents and frequency of administration, patients can develop both acute and delayed CINV. According to the most recent guidelines from major medical organizations such as National Comprehensive Cancer Network and American Society of Clinical Oncology, the use of 5-hydroxytryptamine3 (5-HT_3_; serotonin) receptor antagonist, in combination with and neurokinin1 receptor antagonist (aprepitant) and the corticosteroid dexamethasone is recommended in patients receiving moderately emetogenic chemotherapy [4,5]. In the case of highly emetogenic therapy, a four-drug regimen is recommended, including 5-HT_3_ receptor antagonist, neurokinin1 receptor antagonist, dexamethasone and the antipsychotic drug olanzapine [4,5]. To date, the most commonly used 5-HT_3_ receptor antagonists are granisetron and ondansetron (first-generation 5-HT_3_ receptor antagonists) and palonosetron (second-generation 5-HT_3_ receptor antagonists) [3,5]. While granisetron and ondansetron have significant efficacy in the prevention of acute CINV (0–24 h after chemotherapy) in cancer patients receiving moderately and highly emetogenic chemotherapy, palonosetron has a higher potency and efficacy for the management of delayed CINV (after 24–120 h after chemotherapy) [3,6,7]. When combined with 5-HT_3_ receptor antagonist and dexamethasone, the neurokinin1 receptor antagonist aprepitant prevents CINV in patients receiving highly emetogenic chemotherapy [8]. This four-drug regimen is recommended for the control of both acute and delayed CINV [3,9].

The concept that 5-HT_3_ and its receptors are involved in the emetic reflex was revealed in several earlier studies [10,11]. In addition to the classic 5-HT_3_ receptor antagonists, there are many natural compounds derived from plants which inhibit 5-HT_3_ receptors such as the alkaloids cocaine and morphine (for review, [12]), the antimalarial drug quinine [13], cannabinoids, nicotine and terpenes [12,14,15]. In fact, there has been an increasing tendency towards using natural plant products as important therapeutic entities in the treatment of different illnesses. As such, these agents have advantages over synthetic drugs because they have fewer side effects (such as headache and constipation) in patients undergoing CINV [16] and are more cost-effective [17].

Allosteric modulators alter the receptor conformation by binding to a site distinct from the endogenous agonist binding site (orthosteric site) [18]. As a result, the binding and/or functional properties of agonist binding to orthosteric site may be affected [19]. Negative allosteric modulators decrease the efficacy of the endogenous receptor agonist without inducing complete receptor inhibition caused by orthosteric inhibitors and therefore, maintain the native pattern of the receptor activation largely intact [18,20,21]. Compared to homomeric receptors, the heteromeric 5-HT_3_ receptor contains an increased number of potential allosteric sites for drug interaction. As allosteric sites exhibit greater structural diversity than orthosteric sites, they are more likely to allow selective targeting by modulators than orthosteric sites [18,20,21]. In this article, we examine the results of earlier studies on the actions of natural negative allosteric modulators of 5-HT_3_ receptors. Natural negative allosteric modulators of 5-HT_3_ receptors could be used as useful therapeutic agents in the treatment of CINV in the future.

## 2. 5-hydroxytryptamine3 Receptors

5-HT_3_ receptors represent one of the seven families of serotonin receptors (5-HT_1–7_). They are members of the Cys-loop ligand-gated ion channel family and therefore, they differ structurally and functionally from the other six classes of G-protein coupled serotonin receptors [22]. The functional 5-HT_3_ receptor is a pentamere of five subunits that surround a water-filled ion channel [23,24]. Each subunit is made of 3 domains: (1) An extracellular domain that forms the ligand-binding site, (2) A transmembrane domain consisting of four membrane-spanning helices (M1–M4) that facilitate ion movement across the membrane, and (3) An intracellular domain formed by the large M3–M4 intracellular loop, which is responsible for receptor modulation, sorting, and trafficking, and contains openings that impact ion conductance [23,24,25]. The endogenous ligand binding site is located in the extracellular domain at the interface of two adjacent subunits, where binding is coordinated by the convergence of six peptide loops [26]. To date, the five different subunits (A–E) of 5-HT_3_ receptor have been cloned but only the A and B subunits have been studied extensively. Moreover, all functional 5-HT_3_ receptors require the presence of at least one A subunit [25].

5-HT_3_ receptors are located in many brain areas and are believed to play important roles in psychiatric disorders such as depression, motility of gastrointestinal system, emesis, neurodevelopment and nociception, [12,25,27]. Like other channels that are permeable to the cations, Na^+^, K^+^, and Ca^2+^ [28,29,30], the 5-HT_3_ receptors mediate fast excitatory depolarizing responses in pre- and post-synaptic neurons in the central and peripheral nervous system [31].

## 3. 5-hydroxytryptamine3 Receptor-Mediated Nausea and Vomiting

The role of 5-HT_3_ receptors in nausea and vomiting has been well-established [32,33]. The mechanism by which nausea and vomiting are triggered involves 5-HT_3_ receptors both in the central nervous system and the gastrointestinal tract [34]. Vomiting is triggered when afferent impulses from the cerebral cortex, chemoreceptor trigger zone and vagal afferent fibers of the gastrointestinal tract travel to the vomiting center, located in the medulla. Efferent impulses then travel from the vomiting centre to the abdominal muscles, cranial nerves, salivation centre, and respiratory centre, causing vomiting [35]. Large numbers of 5-HT_3_ receptors are expressed by enteric sensory neurons in the mucosal layer and in the nerve cell body of interneurons and motor neurons in the enteric nervous system [36]. It has been suggested that vomiting occurs because of the stimulation of enterochromaffin cells of the intestinal mucosa which results in the release of 5-HT and subsequent stimulation of peripheral 5-HT_3_ receptors of ganglionic and synaptic transmission in the myenteric plexus. This action, along with the local release of 5-HT in the area postrema, located on the dorsal surface of the medulla oblongata, is suggested to trigger the vomiting reflex [37].

Currently, there is a wide range of 5-HT_3_ antagonists available for clinical use. In fact, drugs that selectively antagonize 5-HT_3_ receptors are currently among the most effective antiemetic agents, and therefore are considered the “gold standard” in the treatment of CINV [25,34,37]. However, the wide use of synthetic antiemetic drugs has been accompanied with adverse side effects such as headache, hypotension and extra pyramidal side effects [17]. Furthermore, they are ineffective in up to 30% of CINV cases [16,38], warranting the search for more effective anti-emetics for CINV treatment. In this aspect, plants have drawn significant interest in the field of treatment of CINV as compared to chemical drugs due to having more advantages, fewer side effects in patients undergoing CINV, as well as being more cost-effective [17].

## 4. Natural Negative Allosteric Modulators of 5-hydroxytryptamine3 Receptors

As 5-HT_3_ receptor antagonists were found to be effective antiemetic agents against CINV, much interest was drawn towards identifying agents that are capable of affecting/modulating the function of 5-HT_3_ receptors [11,39]. In the below sections, we review the effects of natural negative allosteric modulators of 5-HT_3_ receptors.

### 4.1. Terpenes

**Figure 1:**
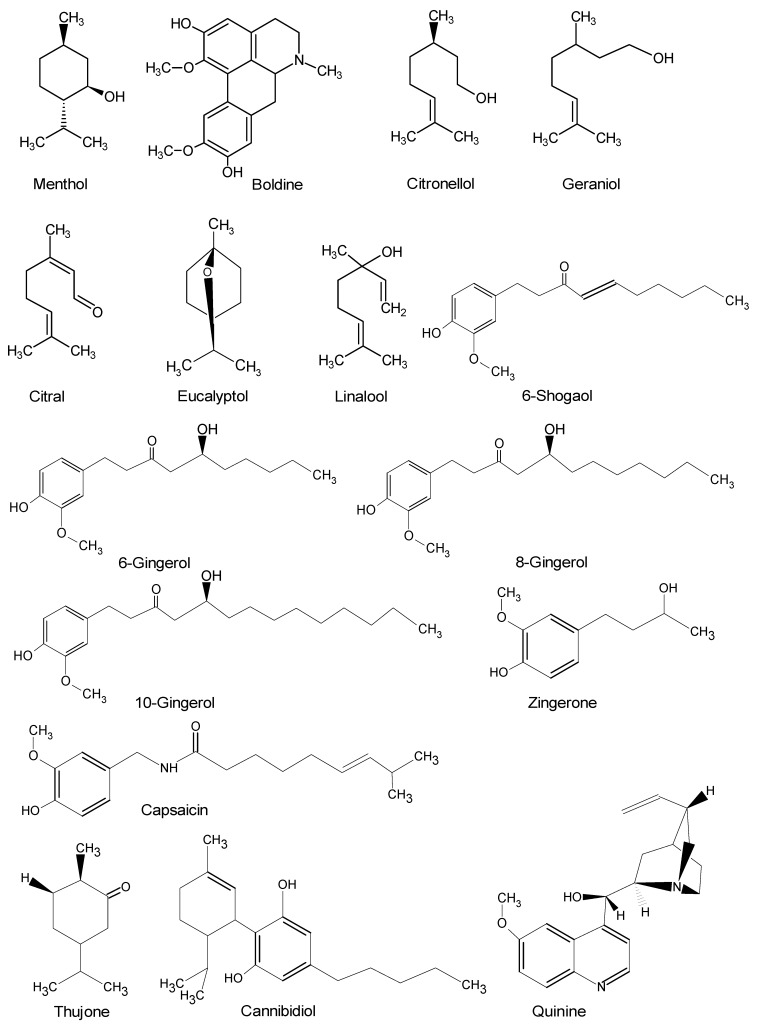
Natural negative allosteric modulators of 5-HT_3_ receptor.

Menthol is a monocyclic terpene alcohol (Figure 1) and a natural product of the peppermint plant *Mentha × piperita* (Lamiaceae). It is commonly used as part of analgesic, antiseptic, topical antipruritic, and cooling formulations. In addition, it is widely used as a natural product in cosmetics, a flavoring agent, and as an intermediate in the production of other compounds. Menthol-containing medications are currently available for a number of conditions, including respiratory diseases, gastrointestinal disorders, common cold, and musculoskeletal pain [40,41,42].

Several studies have reported the potential modulatory effect of menthol on the 5-HT_3_ receptor [15,43,44,45] (Table 1). An earlier study conducted by Heimes et al., 2011 used three in vitro models to investigate the effects of peppermint oil and its active constituent, menthol, on 5-HT_3_ receptors. The models included [^14^C] guanidinium influx into N1E-115 cells which express 5-HT_3_ receptors, isotonic contractions of ileum isolated rat and equilibrium competition binding studies using GR65630 (3-(5-methyl-1H-imidazol-4-yl)-1-(1-methyl-1H-indol-3-yl)-1-propanone, a radioactively-labeled 5-HT_3_ receptor antagonist. Both peppermint oil and menthol, inhibited the serotonin-induced flux of [^14^C] guanidinium into N1E-115 cells that express 5-HT_3_ receptors in a concentration-dependent manner. Furthermore, both compounds were able to reduce serotonin-induced contraction of the ileum isolated from rat. Interestingly, peppermint oil and menthol did not compete for the binding site on 5-HT_3_ receptor, which suggests that they bind to an allosteric binding site that is distinct from the binding site of serotonin [44].

The effects of menthol on the functional properties of human 5-HT_3A_ receptors expressed in *Xenopus laevis* oocytes have been studied by Ashoor et al. [43]. Menthol, at an IC_50_ value of 163 µM, caused an inhibition of the 5-HT-induced inward current. The menthol effect was concentration-dependent and was G-protein-independent as GTPγS activity was unchanged. Furthermore, pretreatment with G_i_ and G_o_ inhibitor, pertussis toxin, did not alter the inhibitory effect of menthol. Furthermore, menthol actions were not stereo-selective as (+), (−) and racemic menthol inhibited the currents mediated by 5-HT_3_ receptor in the same manner [43]. Supporting the data observed by Heimes et al. [44], menthol did not affect the binding of the orthosteric 5-HT_3_ receptor antagonist, GR65630 the 5-HT binding site. In addition, increasing the concentration of 5-HT did not alter the inhibitory effect of menthol, which indicates that menthol acts as an allosteric modulator of 5-HT_3_ receptor as a non-competitive antagonist. Interestingly, menthol has also been shown as a negative allosteric inhibitor of α_7_ [46] and α_4_β_2_ [47,48] nicotinic acetylcholine receptors. Apart from its effect on 5-HT_3_ and nicotinic acetylcholine receptors, menthol was also found to activate TRPM8 receptors [49,50] and receptors for inhibitory neurotransmitters, including GABA_A_ and Glycine [51,52,53,54] (Table 2).

Walstab et al. investigated the actions of the aporphine alkaloid boldine and menthol isomers on human recombinant homomeric 5-HT_3A_ and heteromeric 5-HT_3AB_ receptors expressed in HEK293 cells utilizing luminescence-based Ca^2+^ assay, membrane potential assay and radioligand binding assay. Both compounds inhibited the 5-HT-induced activation of 5-HT_3_ receptors at micromolar concentrations. Boldine was a more potent inhibitor to 5-HT_3A_ compared to 5-HT_3AB_ receptors. Interestingly, menthol inhibited both receptors in a non-competitive and stereo-selective manner. (+)-menthol had less potent inhibitory action compared to the (−)-menthol which was 11-fold potent towards the homomeric 5-HT_3A_ receptor [15].

In a recent study, the actions of a number of terpenes and pungent substances on human 5-HT_3A_ receptors recombinantly expressed in *Xenopus laevis* oocyte were tested [45]. The results of this study have shown that 5-HT_3A_ receptors are inhibited by a variety of terpenes and pungent substances with some of them belonging to the vanilloid class. Within the acyclic monoterpenes tested, citronellol and geraniol, which are known as the main constituents of *Rosa damascena* flower essential oils [96], were the most potent blockers for 5-HT_3A_ receptors. Citronellol decreased the 5-HT_3A_ receptor-mediated currents with an IC_50_ of 64 µM. Geraniol was less potent compared to citronellol with an IC_50_ of 188 µM. (−)-Menthol also decreased the 5-HT_3A_ receptor-mediated currents but with lower potency than citronellol (IC_50_ 489 µM) [45]. As citronellol and geraniol have structures similar to menthol, further studies are warranted to test if these compounds utilize the same binding site as menthol on the 5-HT_3A_ receptors.

The effects of terpenoids on 5-HT_3_ receptors were also recently studied by Jarvis et al. [97]. The terpenoids citral, eucalyptol, and linalool were tested for their effects on the electrophysiological and binding properties of human 5-HT_3_ receptors expressed in Xenopus oocytes and HEK293 cells, respectively [97]. All of the terpenoids inhibited 5-HT_3_ receptors with IC_50_ values in the µM range (citral IC_50_ = 120 µM; eucalyptol IC_50_ = 258 µM; linalool IC_50_ = 141 µM) [97]. The IC_50_ values were also comparable to those for similar terpenoid compounds, such as menthol (163 µM) [43].

In addition, all the tested compounds non-competitively inhibited the maximal 5-HT in a concentration-dependent manner and did not compete with the fluorescently-labelled antagonist granisetron. Homology modeling and ligand docking predicted the binding to a transmembrane cavity at the interface of adjacent subunits. This can be explained by the lipophilic nature of these compounds. In fact, the results are consistent with the slow wash-in and wash-out that is observed as the compounds diffuse into the membranes before reaching their target [55]. Collectively, the effects of terpenoids on 5-HT_3_ receptors can be added to the increasing list of structurally related natural plant compounds that modulate voltage- and ligand-gated ion channels [15,45,46,51,98,99,100,101].

### 4.2. Ginger Constituents

The rhizome of *Zingiber officinale* Roscoe (Zingiberaceae), commonly known as ginger, has been used for centuries in treating pregnancy-induced nausea and vomiting [102,103]. The anti-emetic effect of ginger and its active constituents have been investigated in earlier studies to establish the mechanism for their antiemetic activity [12,104,105]. The best characterized constituents of ginger are the pungent substances gingerols and shogaols [56,106]. In particular, the compounds 6-, 8-, 10-gingerol and 6-shogaol were shown in different in vivo studies to be, at least, partly responsible for the anti-emetic properties of ginger [107,108]. In this regard, Abdel-aziz et al. [106] used three different in vitro models to investigate the effects of ginger extracts on 5-HT_3_ receptors: 1) [^14^C] guanidinium influx into N1E-115 cells which express 5-HT_3_ receptors, 2) isotonic contractions of the isolated guinea-pig ileum induced by the highly selective HT_3_ receptor agonist SR57227A ((4-amino)-(6-chloro-2-pyridyl)L-piperidine hydrochloride), and 3) equilibrium competition binding studies using a radioactively labeled 5-HT_3_ receptor antagonist ([^3^H]GR65630). All tested ginger extracts caused concentration-dependent inhibition of [^14^C] guanidinium influx through 5-HT_3_ receptor channels as well as contractions of the guinea-pig ileum induced by SR57227A. The order of potency for both models was: 6-shogaol ≥ 8-gingerol 10-gingerol ≥ 6-gingerol. These compounds did not displace the 5-HT_3_ antagonist [^3^H]GR65630 from the ligand binding site neither on intact N1E-115 cells nor on the purified membranes of HEK-293 cells over-expressing the h5-HT_3_ receptor indicating that they are non-competitive inhibitors of 5-HT_3_ receptors [106]. Similarly, in the guinea pig myenteric plexus preparation (bioassay for contractile 5-HT_3_ receptors), the 5-HT maximal responses were depressed by 10-gingerol and 6-shagoal. 10-gingerol decreased the response to 5-HT from 93% to 65 % at an antagonist concentration of 3 µM and to 48% at an antagonist concentration of 5 µM. 6-Shogaol (3 µM) was less potent compared to 10-gingerol and induced depression to 61% at an antagonist concentration of 3 µM. It is concluded that the efficacy of ginger in reducing nausea and vomiting may be based on the inhibitory effect of gingerols and shogaols at 5-HT_3_ receptors [56].

In support of the above study, Walstab et al. [109] showed that ginger extracts and its pungent constituents inhibited the activation of human 5-HT_3A_ and 5-HT_3AB_ receptors heterologously expressed in HEK293 cells in a concentration-dependent manner [109]. Furthermore, with increasing concentration of pungent compounds, ginger extracts inhibited both receptors, confirming that they are part of ginger’s active principle. Inhibition potencies of 6-gingerol and 6-shogaol on both receptors were in the low µM range. The non-competitive inhibition of 5-HT_3A_ and 5-HT_3AB_ receptors by ginger extract was confirmed by [^3^H]GR65630 binding, showing that the ginger extract did not displace the radioligand from 5-HT_3A_ and 5-HT_3AB_ receptors.

In addition to the previously mentioned ginger extracts, Jin et al. reported that zingerone is a non-competitive inhibitor of 5-HT_3_ on visceral afferent neurons [58]. Using patch-clamp methods, they showed that zingerone inhibited the 5-HT response in a manner similar to the pungent substances 6-shogaol and 6-gingerol. The order of inhibitory potency for these compounds were 6-shogaol >6-gingerol >zingerone. The IC_50_ of 6-shogaol and 6-gingerol were 128 and 39 times lower than zingerone, respectively, indicating that 6-shogaol and 6-gingerol are more effective than zingerone. Unlike the competitive 5-HT_3_ receptor antagonist ondansetron, all tested ginger constituents acted as non-competitive antagonists, suggesting that ginger and its pungent constituents exert antiemetic effects by blocking 5-HT-induced emetic signal transmission in vagal afferent neurons [58]. Interestingly, zingerone is the least pungent component of *Zingiber officinale* and is absent in fresh ginger. However, gingerols is converted to zingerone upon cooking or heating. As a consequence, this process reduces the 5-HT_3A_ blocking activity of ginger [57]. Although all data showed that ginger-derived molecules act allosterically, in silico techniques showed that these compounds can also bind to the orthosteric binding site causing competitive inhibition as well [110]. Ginger extracts were also tested against other ion channels such as L-type Ca^2+^, Na^+^ and K^+^ channels. While gingerol caused a dose-dependent inhibition of L-type Ca^2+^ channels in longitudinal mycocytes of rats, both 6-gingerol and 6-shagaol inhibited the generation of action potential in the sensory neurons of rats by inhibiting Na^+^ and K^+^ channels [80].

### 4.3. Capsaicin

Capsaicin, from chili pepper, is another pungent substance that has shown effectiveness in reversing emesis in chemotherapy and operation-induced nausea and vomiting [111,112] and in *Cannabis* hyperemesis syndrome [113,114]. Capsaicin is chemically-related to other vanilloid substances such as gingerols and shogaols. Capsaicin was also found to inhibit 5-HT_3_ receptor expressed in *Xenopus laevis* oocytes [45]. When co-applied with 5-HT (5 µM), capsaicin (100 µM) significantly decreased 5-HT-induced currents up to 52% compared to 83% for 8-gingerol, 62% for 6-gingerol, 42% for 6-shogaol, and 65% for polygodial, the hot substance of dorrigo, mountain pepper or black pepper. The concentration-inhibition curves showed that 8-gingerol, 6-gingerol, capsaicin and polygodial blocked 5HT-induced inward currents in a concentration-dependent manner with IC_50_ values of 40 µM, 46 µM, 98 µM and 71 µM, respectively [45]. Other ion channels were also found to be modulated by capsaicin. In fact, capsaicin was found to modulate both K^+^ and Ca^2+^ channels in Xenopus embryo spinal neurons [45].

### 4.4. Eugenol and Vanilin

In addition to the previously mentioned compounds, other agents such as eugenol and vanillin were also found to modulate the activity of 5-HT_3_ receptors. Both agents showed a non-competitive antagonistic activity on 5-HT_3_ receptors expressed in *Xenopus laevis* oocytes, however, very high concentrations of both compounds were needed to inhibit 5-HT response (IC_50_ 1159 µM for eugenol and IC_50_ 4744 µM for vanillin) making them less effective compared to the other antagonists [45]. Eugenol could also inhibit the actions of different isoforms of T-type Ca^2+^ channels [83] and activate the inhibitory receptor GABA [85,86].

### 4.5. Thujone

The monoterpene thujone is chemically related to menthol and found in plants such as wormwood, thyme and sage [19]. Similar to menthol, thujone has been shown to non-competitively suppress the function of 5-HT_3_ receptors. Using patch clamp technique, Alpha-thujone was found to cause a significant inhibition of both homomeric and heteromeric 5-HT_3_ receptors expressed in HEK293 cells. However, the effective concentration was typically in the high µM range [115]. Furthermore, other ligand-gated ion channels were reported to be sensitive to thujone, including GABA_A_ receptors and α7-nicotinic receptors. Thujone caused a non-competitive inhibition of both receptors expressed in HEK 293 cells and Xenopus oocytes, respectively [94].

### 4.6. Cannabidiol

Previous studies showed that both 5-HT_3_ receptor antagonists and cannabinoids produce antiemetic effects [32,59]. In fact, dronabinol and nabilone which are synthetically produced from Δ^9^-tetrahydrocannabinol, the main psychoactive constituent of *Cannabis sativa*, were approved by the United States FDA for use in CINV refractory to conventional antiemetic therapy [32,59,116]. The limitation of the therapeutic use of THC and its other chemical analogs is the potential development of psychoactive effects through cannabinoid receptors (CB1) present in the central nervous system.

The compound cannabidiol is one of the most abundant cannabinoids of *Cannabis* plant with reported antioxidant, anti-inflammatory, and antiemetic effects. Furthermore, this compound lacks any psychoactive properties due to the low affinity for the cannabinoid receptors, CB1 and CB2 [117,118,119]. Thus, pharmaceutical interest in this compound has increased significantly in recent years [19,117,119,120]. The effect of cannabidiol on the function of 5-HT_3A_ receptors expressed in *Xenopus laevis* oocytes was investigated using two-electrode voltage-clamp techniques. Cannabidiol was capable of reversing 5-HT-evoked currents in a concentration-dependent manner, with an IC_50_ = 0.6 µM [121]. Although the potency of the 5-HT was not altered, its efficacy was significantly decreased by cannabidiol, indicating that cannabidiol did not compete with the 5-HT binding site on the receptor. In agreement with these findings, radioligand binding studies indicated that displacement of [^3^H]GR65630 by 5-HT was not significantly altered by cannabidiol, further suggesting that the compound does not bind with 5-HT binding site. These findings indicate that cannabidiol acts as an allosteric modulator of 5-HT_3_ receptor. Allosteric modulation by cannabidiol has also been reported for several structurally different ion channels [117]. Cannabidiol caused a noncompetitive inhibition of the α7-nicotinic receptors [91] in a manner similar to its action on the 5-HT_3A_ receptors, while it activated glycine receptors and TRPA1 channels [85,86].

### 4.7. Other Compounds

There are other natural compounds derived from plants which also target 5-HT_3_ receptors such as the alkaloids cocaine and morphine (for review [60], both of which are potent competitive inhibitors of 5-HT_3_ receptors (for review, [12]). Interestingly, quinine, the antimalarial drug, is a competitive inhibitor of 5-HT_3A_ receptors whereas it non-competitively inhibits 5-HT_3AB_ receptors with a tenfold less potency [13]. Local anesthetics such as lidocaine and bupivacaine, are also competitive and allosteric antagonists at the 5-HT_3A_ receptor [122].

In the current article, we have reviewed the effects of natural negative allosteric compounds on 5-HT_3_ receptors according to different preparations and a wide variety of techniques, ranging from two-electrode voltage clamp to binding assays. Unlike 5-HT_3_ receptor selective antiemetic drugs which bind at the 5-HT binding site, all the compounds reviewed in this article inhibit the 5-HT_3_ receptor by binding to a modulatory binding site distinct from the 5-HT recognition site. These compounds did not compete with or displace the 5-HT_3_ antagonist, as evident in electrophysiological and ligand binding studies. The therapeutic potential of allosteric ligands stems from their ability to modulate receptor function while maintaining the native pattern of the receptor activation largely intact [20,21]. By decreasing the efficacy of endogenous 5-HT_3_ receptor agonist, negative allosteric modulators of 5-HT_3_ receptors can act as fine-tuning tools that may not affect physiological conditions, but may be very active in pathophysiological states such as CINV, without causing complete receptor inhibition [12]. This would lead to better safety profile compared to competitive antagonists. Since allosteric sites exhibit greater structural diversity than orthosteric sites [18], they are more likely to allow selective targeting by natural modulators.

In conclusion, the identification of allosteric binding sites for natural plant extracts and compounds is expected to possess a promising outcome in developing novel anti-emetics lacking the typical side effects caused by setrons [106]. Considering the reported effects of allosteric modulatory compounds, the development of novel antiemetic drugs based on naturally produced compounds may be possible.

## Figures and Tables

**Table 1:** Summary of natural negative allosteric inhibitors of 5HT_3_ receptor.

Compound	Concentration	Preparation	References
Menthol	>100 µM	[^14^C] guanidinium influx into N1E-115 cells. Isotonic contractions of the ileum isolated rat. Equilibrium competition binding studies.	[44]
	IC_50_ = 163 µM	*Xenopus laevis* oocytes	[43]
	IC_50_ = 4.75 µM IC_50_ = 489 µM	HEK293 cells *Xenopus laevis* oocytes	[15]
			[45]
Boldine	IC_50_ = 5.94 µM	HEK293 cells	[15]
Citronellol	IC_50_ = 64.3 µM	*Xenopus laevis* oocytes	[45]
Geraniol	IC_50_ = 188 µM	*Xenopus laevis* oocytes	[45]
Citral	IC_50_ = 120 µM	*Xenopus laevis* oocytes and HEK293 cells	[55]
Eucalyptol	IC_50_ = 258 µM	*Xenopus laevis* oocytes and HEK293 cells	[55]
Linalool	IC_50_ = 141 µM	*Xenopus laevis* oocytes and HEK293 cells	[55]
6-shogaol	100 µM IC_50_ = 3.2–10 µM	[^14^C] guanidinium influx into N1E-115 cells. Equilibrium competition binding studies. Isotonic contractions of the isolated guinea-pig ileum. Visceral afferent neurons and HEK293 cells.	[56,57]
8-gingerol	1–3 µM 100 µM	[^14^C] guanidinium influx into N1E-115 cells. Equilibrium competition binding studies. Isotonic contractions of the isolated guinea-pig ileum.	[56]
6-gingerol	88 µM 100 µM IC_50_ = 9–30 µM	[^14^C] guanidinium influx into N1E-115 cells. Equilibrium competition binding studies. Isotonic contractions of the isolated guinea-pig ileum. Visceral afferent neurons and HEK293 cells	[56,57,58]
10-gingerol	IC_50_ = 9–15 µM	[^14^C] guanidinium influx into N1E-115 cells. Equilibrium competition binding studies. Isotonic contractions of the isolated guinea-pig ileum.	[56]
Zingerone	IC_50_ = 1.19 mM	Visceral afferent neurons	[58]
Capsaicin	IC_50_ = 98.1 µM	*Xenopus laevis* oocytes	[45]
Eugenol	IC_50_ = 1159 µM	*Xenopus laevis* oocytes	[45]
Vanillin	IC_50_ = 4744 µM	*Xenopus laevis* oocytes	[45]
Thujone	-	HEK293 cells	[59]
Cannabidiol	IC_50_ = 0.6 µM	*Xenopus laevis* oocytes	[60]
Quinine	IC_50_ = 1.06 µM	*Xenopus laevis* oocytes	[13]

**Table 2:** Summary of the effects of natural negative allosteric inhibitors of 5HT_3_ on other target proteins.

Compound	Target protein	Effect	References
Menthol	GABA_A_	Potentiation	[52,53,54]
	Glycine	Potentiation	[51]
	Nicotinic receptors	Reduction Up-regulation	[47,61,62] [63]
	TRP channels	Potentiation	[49,50]
	Na^+^ channels	Blocking	[64]
	Ca^2+^ channels	Inhibition	[65,66,67]
	K^+^ channels	Activation	[68]
	TRP channels	Activation Inhibition	[49,50,69] [70]
Boldine	TRP channels	Inhibition	[71]
Citral	TRP channels	Activation	[72]
Eucalyptol	Na^+^ channels	Inhibition	[73]
	TRP channels	Activation	[74,75,76]
Linalool	Na^+^ channels	Inhibition	[77]
	Nicotinic receptors	Reduction	[78]
	TRP channels	Activation	[74,79]
Gingerol	L-type Ca^2+^ channels	Inhibition	[80]
6-gingerol	Na^+^ channels	Blockage	[81]
	K^+^ channels	Inhibition	[82]
6-shogaol	Na^+^ channels	Blockage	[81]
Capsaicin	K^+^ channels	Inhibition	[82]
Eugenol	T-type Ca^2+^ channel	Inhibition	[83,84]
	GABA_A_ receptors	Activation	[85,86]
	K^+^ channels	Inhibition	[87,88]
Cannabidiol	TRP channels	Activation	[74,89,90]
	α_7_-nicotinic receptors	Inhibition	[91]
	Glycine receptors	Activation	[92]
Thujone	TRP channels	Activation	[93]
	α_7_-nicotinic receptors	Inhibition	[94]
	GABA_A_ receptors	Inhibition	[95]
